# Downregulation of protein kinase CK2 induces autophagic cell death through modulation of the mTOR and MAPK signaling pathways in human glioblastoma cells

**DOI:** 10.3892/ijo.2012.1635

**Published:** 2012-09-21

**Authors:** BIRGITTE B. OLSEN, TINA H. SVENSTRUP, BARBARA GUERRA

**Affiliations:** 1 Department of Nuclear Medicine, Odense University Hospital, 5000 Odense;; 2 Department of Biochemistry and Molecular Biology, University of Southern Denmark, 5230 Odense, Denmark

**Keywords:** glioblastoma cells, autophagy, CK2, mammalian target of rapamycin, extracellular signaling-regulated protein kinase 1/2, reactive oxygen species

## Abstract

Glioblastoma multiforme is the most common primary brain tumor and one of the most aggressive types of cancer in adults. Survival signaling and apoptosis resistance are hallmarks of malignant glioma cells. However, recent studies have shown that other types of cell death such as autophagy can be induced in malignant glioma cells. This suggests that stimulation of this process may be explored in new therapeutic strategies against glioblastoma multiforme. Protein kinase CK2 is a highly conserved and constitutively active enzyme that promotes numerous cellular processes such as survival, proliferation and differentiation. CK2 has been found elevated in several malignancies including brain tumors, and to confer resistance against chemotherapeutic agents and apoptotic stimuli. Recently, we have shown that the siRNA-mediated downregulation of CK2 leads to cell death in DNA-PK-proficient human glioblastoma cells. We show, here, that lack of CK2 results in significant induction of autophagic cell death in two human glioblastoma cell lines, M059K and T98G, as indicated by the positive staining of cells with the acidotropic dye acridine orange, and the specific recruitment of microtubule-associated protein 1 light chain 3 (LC3) to autophagosome membranes. Induction of autophagy is accompanied by CK2-dependent decreased phosphorylation of p70 ribosomal S6 and AKT kinases and significantly reduced expression levels of Raptor. In contrast, phosphorylation and activity levels of ERK1/2 are enhanced suggesting an inhibition of the PI3K/AKT/mTORC1 and activation of the ERK1/2 pathways. Furthermore, siRNA-mediated silencing of CK2 results in increased mitochondrial superoxide production in both glioblastoma cell lines. However, mitochondrial reactive oxygen species release correlates with induction of autophagy only in T98G cells. Taken together, our findings identify CK2 as a novel component of the autophagic machinery and underline the potential of its downregulation to kill glioblastoma cells by overcoming the resistance to multiple anticancer agents.

## Introduction

Glioblastoma multiforme is the most common primary brain tumor in humans arising from cells of the glia lineage. The name denotes a very heterogeneous type of tumor with respect to cell morphology and chromosome aberrations, which includes extended deletions, gain of entire chromosomes or chromosome arms and gene amplification (1). Surgical resection and radiotherapy are the first and second stage of treatment of glioblastoma, respectively, while the addition of chemotherapy to radiation has so far shown limited improved survival (2,3). In mammals, exposure of cells to radiation causes DNA double-strand breaks that are generally repaired via non-homologous end joining following activation of DNA-dependent protein kinase (DNA-PK) (4). However, when DNA damage is excessive, cells induce DNA-PK-mediated apoptosis. It has been reported that exposure to high doses of ionizing radiation leads to autophagy induction in some types of cancer cells including malignant glioma cells (5).

Macroautophagy (hereafter referred to as autophagy) is a biphasic process characterized by the formation of double-membrane vesicles called autophagosomes (i.e. the formation phase) often containing subcellular organelles, which fuse with lysosomes. In the following maturation phase the vesicle content is degraded generating macromolecules and ATP that are recycled as to maintain cellular homeostasis (6). The formation of autophagosomes is mediated by a highly organized and hierarchical team of autophagy-related gene products (ATG proteins) of which microtubule-associated protein light chain 3 (LC3), the mammalian homolog of yeast ATG8p, is the most specific marker as it accumulates on the autophagosomal membrane giving rise to characteristic punctate patterns (6,7). Several recent studies have suggested that autophagy also functions as a pro-death mechanism. In this respect, it has been shown that different types of cancer cells undergo autophagic cell death in response to anticancer therapy (8,9). At present, it remains controversial whether autophagy represents a survival and cytoprotective process or causes death in cancer cells as well as the precise molecular mechanisms that regulate this dynamic process.

Recent studies from our laboratory have revealed that siRNA-mediated downregulation of protein kinase CK2 leads to morphological changes resembling activation of autophagic cells death in human glioblastoma cells (10). Protein kinase CK2 is a constitutively active and highly conserved serine/threonine kinase composed of two catalytic α and/or α′ subunits and two regulatory β subunits. Evidence indicates that the individual subunits do not exist exclusively within the tetrameric complex but also as free proteins (11,12). CK2 expression and activity are deregulated in many human diseases including cancer and while the overexpression often correlates with enhanced cell survival and proliferation, cellular depletion generally results in reduced cell viability and increased cell death, particularly apoptosis (13). Moreover, although mounting evidence underlines the importance of targeting CK2 for activation of apoptosis in cancer cells, the role of this kinase with respect to induction and/or progression of other types of cell death such as autophagy is largely unknown.

In this study, we aimed to shed light on the mechanism by which CK2 silencing induces autophagy in human glioblastoma cells treated with the radiomimetic drug neocarzinostatin. We report evidence that downregulation of protein kinase CK2 results in inhibition of the mammalian target of rapamycin (mTOR) pathway, downregulation of Raptor expression levels and activation of the extracellular signaling-regulated protein kinase 1/2 (ERK1/2) signaling pathway as evidenced by ERK phosphorylation and enhanced kinase activity. The reported results support the notion that CK2 may serve as a potential target for enhancing the cellular response of glioblastoma cells resistant to multiple drug treatments.

## Materials and methods

### Cell culture

The human glioblastoma cell lines M059K and T98G were obtained from the American Type Culture Collection (Rockville, MD, USA). The cells were cultured in DMEM (Invitrogen, Taastrup, Denmark) with 10% FBS and maintained at 37°C in a 5% CO_2_ atmosphere.

### Cell culture and treatments

Cells were transfected with a set of 4 small interfering RNA (siRNA) duplexes directed against CK2α or CK2α′ (On-Target plus SMARTpools, Dharmacon, Lafayette, CO, USA), using Dharmafect I transfection reagent (Dharmacon) for 72 h according to the manufacturer’s instructions. Control experiments were performed transfecting cells with scramble siRNA sequences. Where indicated, cells were incubated with the radiomimetic drug neocarzinostatin for 24 h (NCS, a gift from Dr Hiroshi Maeda, Kumamoto University, Kumamoto, Japan). Endogenous mTOR activity was inhibited by incubating T98G cells with 100 nM rapamycin (Calbiochem, Nottingham, UK) and M059K cells with 50 nM rapamycin, respectively. Maturation of autophagy vacuoles was inhibited by treating cells with 50 nM bafilomycin A (Calbiochem) for 6 h prior to cell analysis.

### Flow cytometry

Autophagy was analyzed by staining cells with the vital dye acridine orange (Sigma, Brondby, Denmark). Briefly, cells were incubated with acridine orange at a final concentration of 1 *μ*g/ml for 15 min prior to trypsinization and flow cytometry analysis on a FACSCalibur (BD Biosciences, San Diego, CA, USA) using CellQuest Pro Analysis Software (BD Biosciences). To detect mitochondrial superoxide production, cells were trypsinized and incubated with 5 *μ*M MitoSOX™ Red Mitochondrial Superoxide indicator (Invitrogen) for 15 min prior to FACS analysis. ROS scavengers treatment was performed by incubating cells with 100 *μ*M BHA or 50 *μ*M BPN (both from Sigma) for 24 h prior to harvesting and subsequent analysis by flow cytometry.

### Western blot analysis

Whole cell extracts and immunoblotting were as previously described (14). The primary antibodies employed in this study were: mouse monoclonal anti-CK2β and -CK2α (both from Calbiochem); rabbit polyclonal anti-CK2α′ obtained by immunizing rabbits with a specific peptide (SQPCADNAVLSSGTAAR) deriving from human CK2α′; mouse monoclonal anti-β-actin (Sigma); mouse monoclonal anti-mTOR and -AKT1 (both from BD Biosciences), rabbit monoclonal anti-Raptor and -ERK1/2 (p-T202/Y204); rabbit polyclonal anti-mTOR (p-S2481) and -ERK1/2 (all from Cell Signaling Technology, Beverly, MA, USA); mouse monoclonal anti-AKT (p-S473) and -p70 S6 kinase (p-T389) (both from Cell Signaling Technology) and rabbit polyclonal anti-p70 S6 kinase (Santa Cruz Biotechnology, Santa Cruz, CA, USA). Protein bands were then visualized by a chemiluminescence detection system following the manufacturer’s guidelines (CDP-Star; Applied Biosystems, Foster City, CA, USA).

### In vitro protein kinase assay

The activity of ERK1/2 was tested using a non-radioactive p44/42 MAPK kinase assay kit (Cell Signaling Technology) following the manufacturer’s instructions. Briefly, whole cells extracts were immunoprecipitated with rabbit monoclonal anti-phospho-p44/42 MAPK (ERK1/2) (Thr202/Tyr204) antibody immobilized on sepharose beads. Immunoprecipitates were subjected to a non-radioactive kinase assay in the presence of 0.25 *μ*g recombinant GST-fused Elk-1, 1X kinase buffer-containing 200 *μ*M ATP. Incubation time was 30 min at 30°C. Phosphorylation of Elk-1 was revealed by labeling western blot membranes with mouse monoclonal anti-phospho-Elk-1 (Ser383) antibody.

### EGFP-LC3 translocation assay

Cells were grown on cover-slips and transfected with CK2-siRNA for 48 h, prior to addition of NCS for 24 h. To visualize GFP-LC3, cells were infected with Premo Autophagy Sensors (LC3B-FP, wild-type) BacMam2.0 (Invitrogen) according to the manufacturer’s instructions. The LC3B (G120A)-FP mutant form was employed in control experiments, as the mutation of the expressed LC3B prevents its cleavage and subsequent lipidation following induction of autophagy. After 24 h, cells were fixed with 4% paraformaldehyde for 20 min, permeabilized with 0.2% Na-citrate/0.2% Triton X-100 for 5 min and counterstained with 4′,6-diamidino-2-phenylindole (DAPI) and analyzed on a DMRBE microscope (×400 magnification) equipped with a Leica DFC420C camera. Pictures were processed using ImageJ software (NIH, Bethesda, MD, USA). For each condition, at least 200 GFP-LC3-expressing cells were analyzed and the percentage of puncta per cell, deriving from the expression of GFP, was determined by using the ‘analyze particles’ function in the ImageJ software.

### Statistical analysis

The statistical significance of differences between the means of two groups was evaluated by the two-tailed t-test and the level of significance was as indicated in the figure legends.

## Results

### Downregulation of protein kinase CK2 induces autophagic cell death in human glioblastoma cells

**Figure 1:**
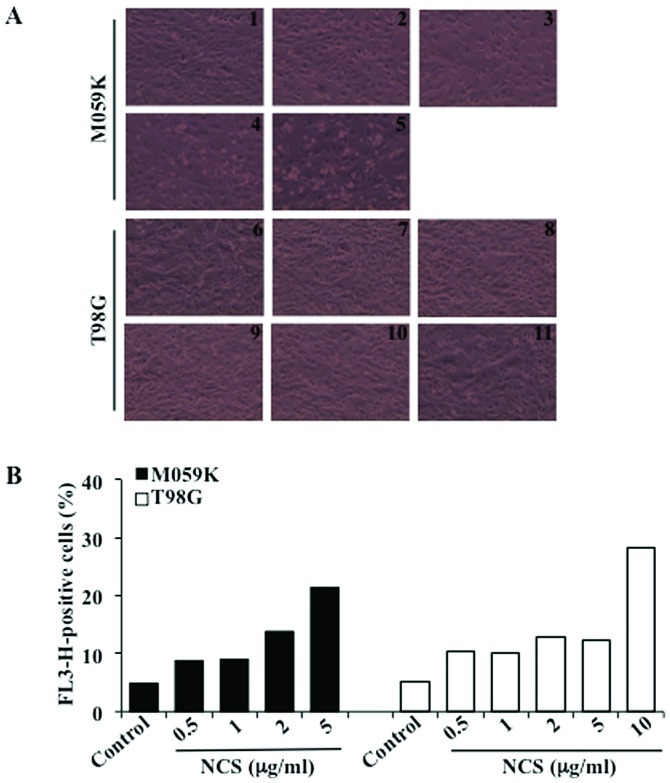
NCS induces autophagy in human glioblastoma cells. (A) Phase-contrast microscopy pictures of M059K and T98G cells left untreated (1 and 6) and incubated with 0.5 *μ*g/ml (2 and 7), 1 *μ*g/ml (3 and 8), 2 *μ*g/ml (4 and 9), 5 *μ*g/ml (5 and 10), 10 *μ*g/ml (11) NCS for 24 h, respectively. Pictures showing morphological changes indicating various degrees of toxicity induced by the aforementioned treatments were taken at ×400 magnification. (B) Cells were treated as described above. Induction of autophagy was determined by flow cytometry analysis of acridine orange-positive cells (FL3-H indicates red-fluorescence intensity). Experiments were repeated three times and data from one representative experiment are shown.

**Figure 2:**
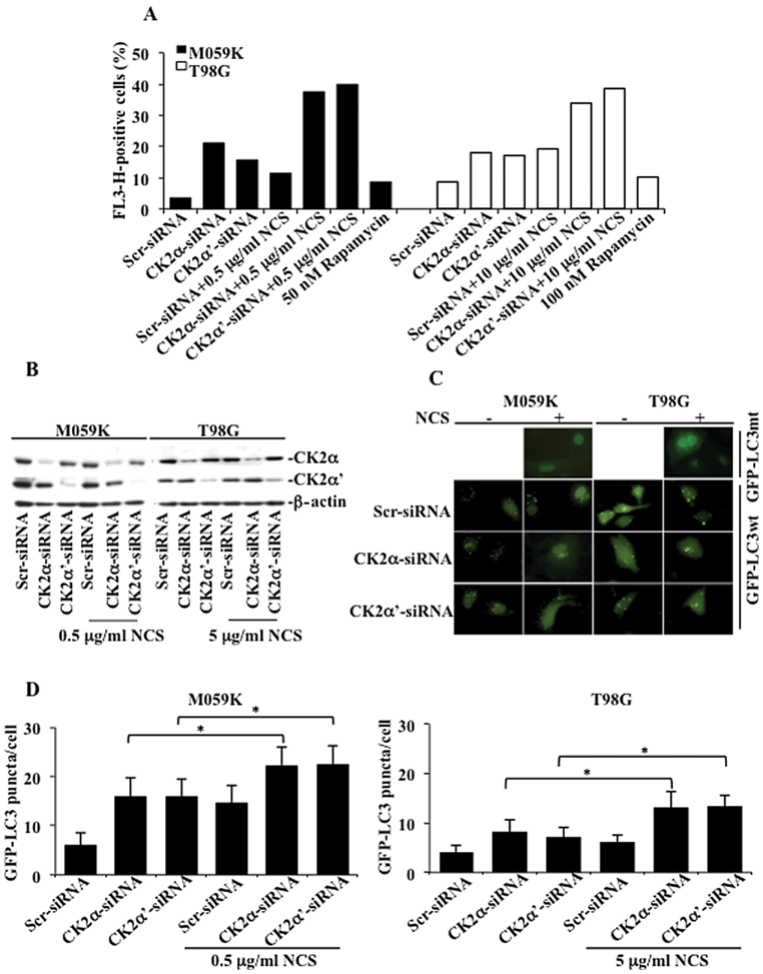
Downregulation of CK2 leads to induction of autophagy. (A) Cells were transfected with scramble siRNA (Scr-siRNA) or with siRNAs against the individual CK2 catalytic subunits for 72 h. Where indicated, NCS was added at the indicated concentrations in the last 24 h of incubation with siRNA. Induction of autophagy was analyzed by flow cytometry as described in Fig. 1. A control experiment is also shown where cells were incubated with rapamycin for 24 h. (B) Western blot analysis of total cell lysates from cells treated as indicated. Proteins were visualized by probing the membranes with antibodies against the indicated proteins. (C) Photomicrographs of cells transfected with Scr-siRNA or CK2-siRNAs for 48 h. In the last 24 h of incubation time, cells were transduced with Premo Autophagy Sensor LC3B-GFP and added 0.5 *μ*g/ml (M059K) and 5 *μ*g/ml (T98G) NCS, respectively. (D) Determination of the average number of puncta, as distinct fluorescence green spots, per cell was performed by analyzing 200 cells per sample. The mean values ± SD are shown. Two independent data set were obtained showing similar results. ^*^P<0.001 indicates statistical significance.

Accumulating evidence indicates that induction of DNA double-strand breaks in some types of cancer cells, including malignant glioma cells, induces autophagic cell death (8,9). To compare the effects of DNA damage on autophagy induction, we analyzed M059K and T98G cells after exposure to the radiomimetic drug neocarzinostatin (NCS) (15). Treatment with increasing concentrations of NCS for 24 h led to marked cytotoxicity in M059K cells when incubated with up to 5 *μ*g/ml NCS while a similar effect was observed in T98G cells exposed to 10 *μ*g/ml NCS (Fig. 1A). Induction of autophagy was determined by flow cytometry after staining of cells with acridine orange, a vital dye which accumulates in acidic compartments emitting bright red fluorescence indicative of autophagic vacuoles formation (AVOs) (16). As shown in Fig. 1B, untreated cells showed negligible cytoplasmic staining as compared to cells treated with increasing concentrations of NCS. NCS (5 *μ*g/ml) led to formation of AVOs in approximately 21% of the M059K cells while a slightly higher percentage (i.e. 28%) was reached with T98G cells when incubated with 10 *μ*g/ml NCS. To assess the role of CK2 in the activation of autophagy, we transfected M059K and T98G cells with a pool of small interfering RNAs (siRNAs) against the individual catalytic subunits of CK2 in the presence or absence of NCS as indicated in Fig. 2A. Cells were subsequently stained with acridine orange and analyzed by flow cytometry. In M059K cells, incubation with NCS resulted in AVOs accumulation in approximately 11% of the total number of cells while reduction of CK2α and -α′ protein levels led to the formation of AVOs in 21 and 15% of the cells, respectively. This percentage increased to approximately 37 and 39% following incubation with NCS, respectively. In the case of T98G cells, the number of cells with AVOs following exposure to NCS or treatment with siRNAs against the CK2 catalytic subunits was approximately 18% while the combined downregulation of the CK2 subunits and treatment with NCS led to up to 38% AVOs-positive cells. Exposure of cells to rapamycin, a known activator of autophagy in malignant glioma cells (17), resulted in the accumulation of AVOs in approximately 10% of the total number of cells.

To confirm the induction of autophagic cell death induced by the siRNA-mediated CK2 silencing, we assessed the presence of a punctate pattern of the green-fluorescent protein (GFP)-tagged LC3 (GFP-LC3) expression. M059K and T98G cells were transfected with siRNAs against the individual catalytic subunits of CK2 for 48 h. Subsequently, cells were transduced with GFP-LC3wt or a mutated form (GFP-LC3mt) to rule out the possibility that expression of GFP-LC3 would lead to an unspecific aggregation rather than its translocation to the autophagosomal membranes (control experiment), and incubated for additional 24 h in the absence and presence of NCS, respectively. Western blot analysis of cell lysates confirmed the significant downregulation of the individual CK2 subunits (Fig. 2B). As shown in Fig. 2C, cells transduced with GFP-LC3mt showed diffuse distribution of GFP-LC3 following incubation with NCS while cells transduced with GFP-LC3wt showed a punctate pattern of GFP-LC3 fluorescence, indicating the presence of autophagic vacuoles and recruitment of LC3 to their surface. Quantification of autophagic-positive cells revealed that downregulation of CK2 increased the incidence of autophagy in both cell types as compared to control experiments (Fig. 2D). Furthermore, when cells were additionally incubated with NCS, the percentage of puncta per cell significantly increased up to 22% in the case of M059K cells and 13% in the case of T98G cells with respect to cells treated with NCS and transfected with Scr-siRNA. Induction of autophagy was lower in T98G than in M059K cells. However, these results indicate that downregulation of CK2 induces autophagic cell death which is significantly enhanced in the presence of NCS and suggest that differences in autophagy stimulation might be related to the intrinsic resistance of different glioblastoma cell types towards induction of this process.

**Figure 3:**
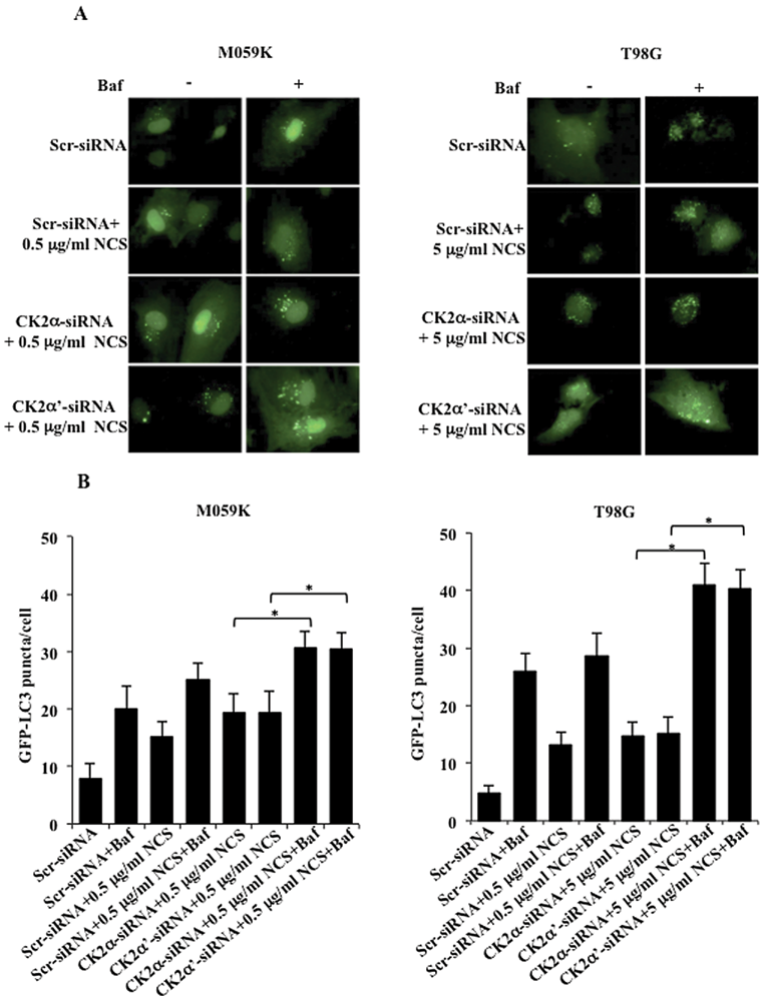
Regulation of autophagic flux by cellular depletion of CK2. (A) Representative fluorescence-based images of cells expressing or lacking CK2 exposed to 50 nM bafilomycin A (Baf) for 6 h before the analysis and NCS, as indicated in the figure, for 24 h before cell analysis. (B) Bar graph indicates the average number ± SD of puncta per cell determined by analyzing 200 cells per sample. Three independent experiments were performed obtaining similar results. ^*^P<0.001 indicates statistical significance.

Accumulation of GFP-LC3 puncta may result from activation of the autophagic process or a defect in the autophagosome maturation. To distinguish between these two events, we induced autophagy in the presence or absence of the vacuolar H^+^-ATP inhibitor bafilomycin A (Baf) which is expected to inhibit the fusion between autophagosomes and lysosomes and, thus, formation of autolysosomes (18). As expected, CK2-depleted cells revealed a punctate pattern of GFP-LC3 following treatment with NCS consistent with data presented above, however, the number of puncta per cell was significantly higher in cells additionally treated with bafilomycin A (Fig. 3A). Determination of the number of puncta per cell indicated that both cell lines depleted of CK2 and treated with NCS and bafilomycin A accumulated a significantly higher number of GFP-LC3-puncta as compared to cells subjected to similar treatment but in the absence of bafilomycin A (Fig. 3B). Flow cytometry analysis of cells stained with acridine orange confirmed data reported above (results not shown). Overall, results reported here suggest that induction of autophagic cell death mediated by silencing of CK2 by siRNA emerges from stimulation of the formation phase of AVOs as indicated by the recruitment of GFP-LC3 to autophagic vacuoles and increased number of puncta per cells upon incubation with bafilomycin A.

### Downregulation of protein kinase CK2 regulates autophagy through the mTOR and ERK1/2 signaling pathways

**Figure 4:**
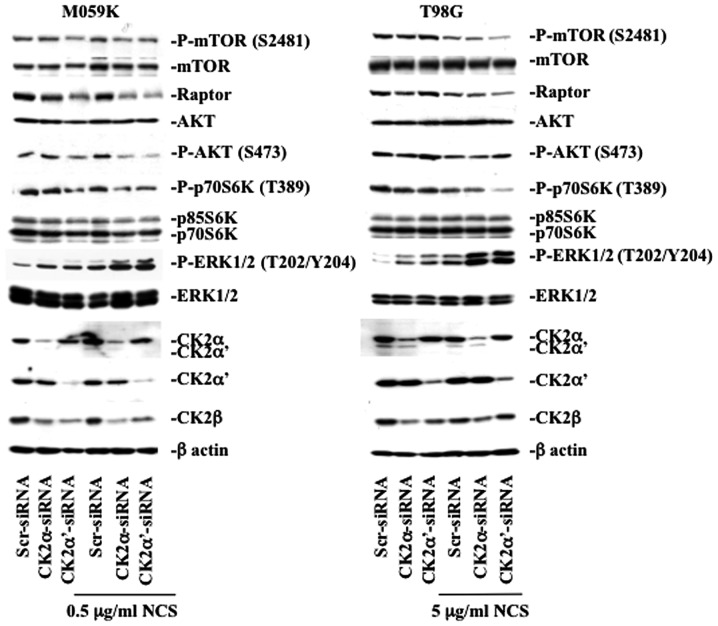
Downregulation of protein kinase CK2 affects the mTOR and ERK1/2 signaling pathways. Cell lysates (30 *μ*g) from cells treated as indicated in the figure were analyzed by western blot analysis using antibodies against the indicated proteins or their phosphorylated form. Anti-β-actin was applied to confirm equal protein loading. At least 4 separate experiments were performed obtaining similar results. Data from one representative experiment are shown.

The class I phosphatidylinositol 3-phosphate kinase (PI3K)/AKT/Raptor-mTOR (mTORC1) signaling pathway and the Ras/Raf/MEK1/2/ERK1/2 pathway are two well-known signaling cascades involved in the regulation of autophagy (19–21). Because of their implication in autophagy regulation, we examined their status upon downregulation of CK2 (Fig. 4). In M059K cells, CK2 depletion led to decreased kinase activity of mTOR reflected by the diminished phosphorylation of mTOR at S2481 which is a marker for autokinase activity *in vivo*(22), decreased phosphorylation of the mTOR downstream target, p70 S6 kinase (p70S6K), and lower AKT phosphorylation at S473. The latter being an important positive regulator of mTOR via pathways involving the TSC1/TSC2 complex (23). Interestingly, downregulation of CK2 led to marked decreased expression levels of Raptor and this effect was particularly pronounced in cells depleted of CK2α′ in the absence or presence of NCS. In T98G cells, the mTOR pathway was mainly affected in CK2α′-siRNA and NCS-treated cells evidenced by the significant decreased mTOR and p70S6K phosphorylation levels and lowered expression of Raptor.

**Figure 5:**
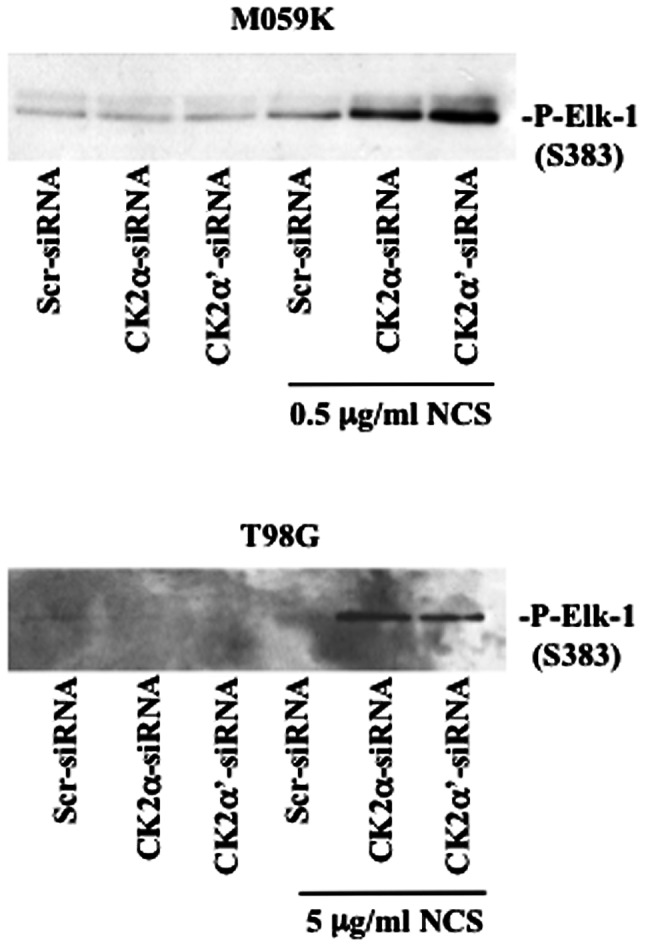
CK2-knockout leads to enhanced ERK1/2 kinase activity in the presence of NCS. Cells were transfected with Scr-siRNA or CK2-siRNAs for 72 h. Where indicated, NCS was added 24 h before harvesting. Whole cell lysates (500 *μ*g) were subjected to a non-radioactive kinase activity assay in the presence of a GST-Elk-1 fusion protein after immunoprecipitation of phosphorylated ERK1/2. The phosphorylation levels of Elk-1 were detected by western blot analysis with an antibody directed against phospho-Elk1-1 (S383). Representative results from two independent experiments are shown.

Next, we examined whether cellular depletion of CK2 affected the phosphorylation of ERK1/2, which is considered a positive regulator of autophagy (24). As predicted, repeated experiments showed that silencing of CK2α and -α′ in the presence of NCS led to marked increase in ERK1/2 phosphorylation in both cell lines, respectively. As CK2 knockdown led to increased phosphorylation of ERK1/2 in cells treated with NCS, we tested whether this effect was associated with increased ERK1/2 kinase activity (Fig. 5). We performed a non-radioactive kinase assay where the activity of endogenous ERK1/2, isolated by immnuoprecipitation, was tested against Elk-1 protein a downstream target of ERK (25). By employing a specific antibody directed against phosphorylated Elk-1, we verified that enhanced phosphorylation of ERK was accompanied by marked kinase activation in cells depleted of CK2 and treated with NCS. Overall, western blot analysis of the aforementioned proteins suggests that induction of autophagy following CK2 knockdown and NCS treatment occurs through inhibition of the PI3K/AKT/mTOR and activation of the ERK1/2 signaling pathways. As cellular depletion of CK2 markedly enhanced the phosphorylation of ERK1/2, we sought to verify whether autophagy would be blocked by ERK inhibition using the mitogen-activated protein kinase kinase1/2 (MEK1/2) inhibitors, U0126 and PD098059 (26) employed in separate experiments. However, autophagy induction was attenuated but not completely suppressed by indirect inhibition of ERK in the presence of the aforementioned MEK1/2 specific inhibitors (results not shown).

### Induction of autophagy mediated by CK2 knockdown is accompanied by the generation of ROS in T98G but not M059K cells

**Figure 6:**
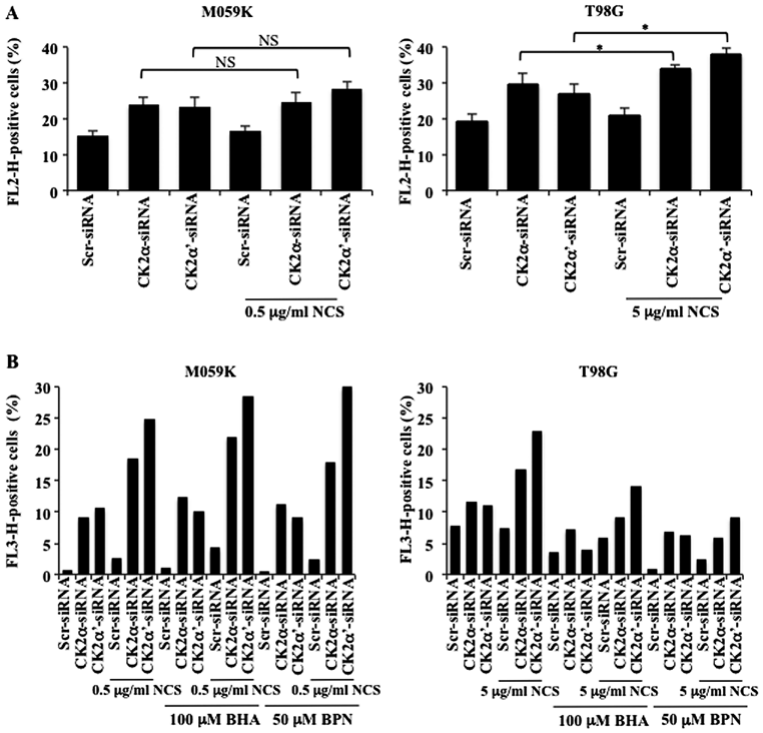
ROS involvement in autophagy induction mediated by CK2-siRNA silencing. (A) Cells were treated as indicated in the figure. Mitochondrial superoxide production was measured in two independent experiments by flow cytometry after labeling cells with MitoSOX™ Red as described in the Materials and methods. FL2-H intensity indicates red-positive cells ± SD expressed in percentage. ^*^P<0.001 indicates statistical significance. NS, not significant. (B) Cells were treated as described above. Where indicated, cells were added 100 *μ*M BHA 24 h before harvesting. Induction of autophagy was determined by flow cytometry of acridine orange-positive cells. At least three independent experiments were performed obtaining similar results. Data from one representative experiment are shown.

Mitochondria are the main source of reactive oxygen species (ROS), which includes superoxide, hydrogen peroxide and hydroxyl radical. It has recently been reported that ROS may regulate several core autophagic pathways (27). When mitochondrial ROS is elevated, the mitochondrial membrane is damaged, resulting in ROS leakage into the cytosol thereby damaging other organelles. Generation of mitochondrial ROS has been shown to correlate with phosphorylation and activation of ERK which co-localizes with mitochondria, AVOs and lysosomes and regulates mitophagy in glioblastoma cells (28). Hence, to test whether induction of autophagy mediated by CK2 downregulation was accompanied by ROS generation, we measured intracellular levels of superoxide, the predominant ROS in mitochondria (29), by flow cytometry after labeling cells with MitoSOX Red reagent a specific mitochondrial superoxide-detecting fluorescent dye. As shown in Fig. 6A, mitochondrial superoxide increased in cells depleted of CK2. Additional treatment with NCS led to slightly higher level of superoxide content which was significant in T98G but not in M059K cells. Because superoxide has been reported to be the major regulator of autophagy among mitochondrial ROS, we investigated whether inhibition of ROS production would be blocked in the presence of ROS scavengers and whether this event would have an influence on autophagy induction. Autophagy was evaluated by flow cytometry of acridine orange stained cells. As shown in Fig. 6B, treatment with butylated hydroxyanisole (BHA) or N-tert-Butyl-α-phenylnitrone (BPN) significantly reduced accumulation of autophagic vacuoles induced by depletion of CK2 in the absence or presence of NCS in T98G cells however, the same effect was not observed in M059K cells. Overall, results reported here indicate that cellular depletion of CK2 is accompanied by ROS production and that the dependency on ROS generation in the regulation of autophagy seems to be cell type-dependent. In this respect, it has been previously reported that terfenadine, a highly potent H1 histamine receptor antagonist, induces autophagy by ROS-dependent and -independent mechanisms in human melanoma cell lines (30).

## Discussion

The process of autophagy is tightly controlled by several signaling cascades and involves various steps including induction, vesicle formation, autophagosome-lysosome fusion, digestion and release of macromolecules in the cytosol. Protein kinases play an important role in the regulation of this process and while some of them such as mTOR, PI3K and AMPK directly regulate components of the autophagic machinery, others, less characterized, may control this process indirectly by influencing level of expression and/or function of autophagy-related proteins. In this study, we demonstrated that downregulation of protein kinase CK2 by RNA interference leads to induction of autophagy in human glioblastoma cells and that the combined exposure to the radiomimetic drug NCS, significantly augments this process. The level of induction of autophagy by CK2-siRNA-mediated silencing was significantly higher than the one induced by rapamycin treatment as shown in Fig. 2. Rapamycin and its derivatives such as CCI-779, RAD001 and AP23573, are well-established inhibitors of mTOR that have been shown to induce modest levels of autophagy potentially because these agents rather than acting as inhibitors of mTOR interfere solely with the function of the mTOR/raptor complex (i.e. mTORC1) and thus, do not block all mTOR forms (31). Accordingly, the fact that CK2 silencing in the absence or presence of NCS treatment led to marked increase in autophagy-positive cells as determined by flow cytometry and analysis of the number of puncta formation in GFP-LC3-positive cells, suggest that CK2 might affect various components/steps of the autophagic process. In this respect, we assessed the expression levels and the activity of a downstream component of the mTORC1 signaling pathway, p70S6K, and found that its phosphorylation was significantly inhibited in both cell lines. In M059K cells, downregulation of CK2α’ alone was sufficient to negatively affect the phosphorylation of p70S6K kinase while this effect was achieved in T98G cells following additional treatment with NCS. This difference might reflect the intrinsic resistance of T98G cells to cell death induction. Analysis of Raptor protein revealed marked downregulation of its expression levels. It remains to be determined the mechanism by which CK2 depletion results in lowered Raptor expression as the regulation might be at the transcriptional and/or translational levels. However, one cannot exclude that the observed reduced phosphorylation of p70S6K, which is a translation regulator, might suppress overall cellular protein synthesis, hence, Raptor expression. On the other hand, decreased phosphorylation of p70S6K seen in CK2 depleted cells, might be a direct consequence of low Raptor expression as it has been demonstrated that the siRNA-mediated downregulation of Raptor leads to reduced phosphorylation of p70S6K and 4E-BP1 (32).

In our study, autophosphorylation of mTOR at S2481 was found repressed. It has recently been demonstrated that, in contrast to what was previously reported, mTORC-associated mTOR S2481 autophosphorylation monitors mTORC intrinsic catalytic activity *in vivo*(33). Hence, our results additionally show that CK2 depletion contributes to the activation of autophagy by blocking the autophosphorylation of mTOR. The two mTOR complexes are linked through AKT since mTORC2 (i.e. mTOR/Rictor)-activated AKT indirectly stimulates mTORC1. We reported that CK2 depletion leads to decreased phosphorylation of AKT at S473. Interestingly, phosphorylation of AKT at S473 has been shown to be upregulated after mTORC1 inhibition. This effect has been proposed to confer resistance to drug treatment (34). The reason for this discrepancy is not entirely clear, however, it suggests that AKT phosphorylation at S473 is subjected to multiple levels of regulation (35,36).

One of the most striking observations that emerged from our studies is the significant increased phosphorylation of ERK1/2 in cells treated with CK2-siRNA and NCS that was accompanied by upregulation of ERK1/2 kinase activity. ERK activity has been associated with constitutive autophagy and autophagic cell death in many cellular models and in response to different stresses including amino acid depletion and various drug treatments (24,37–39). Moreover, direct ERK activation by overexpression of constitutively active MEK has been shown to promote autophagy without any further stimulus (40). Hence, our results support the notion that sustained activation of ERK pathway results in induction of autophagic cell death in glioblastoma cells. The observed failure to reverse completely effects induced by CK2 knockdown in cell treated with the specific MEK1/2 inhibitors, U0126 and PD098059, might depend on the CK2-mediated induction of autophagy through regulation of multiple pathways, i.e. the mTOR and ERK1/2 signaling cascades. This is in accordance to previous data supporting the notion that it is necessary to knockdown more than one of the members that belong to autophagy-related pathways in order to induce this process (41).

An additional observation that emerged from this study is that both cell types showed increased mitochondrial superoxide production following CK2 silencing, however, autophagy induction was affected by treatment with ROS scavengers only in T98G cells. Although it remains to be determined whether mitochondrial ROS generation is a consequence of autophagy activation or a catalyst of this process, the intensity of the death stimulus in the two cell lines might determine the dependency on ROS generation for the induction of autophagy.

**Figure 7:**
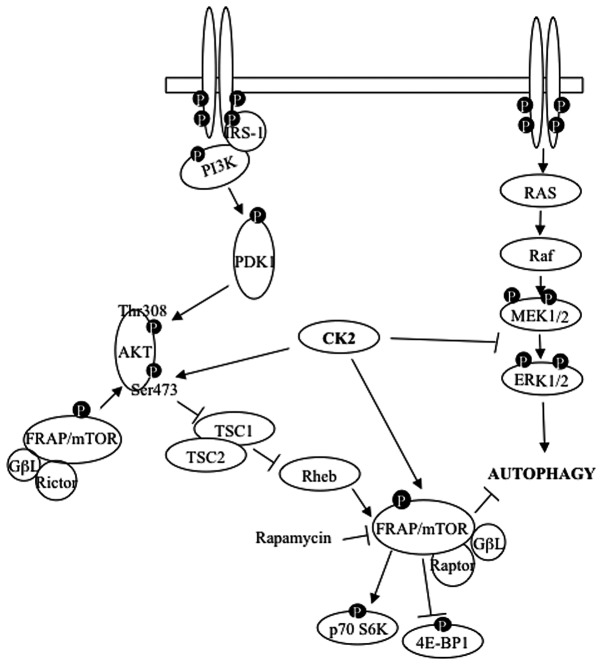
Model of the proposed influence of CK2 on autophagy induction. The model suggests the placement and function of CK2 with respect to the major signaling cascades regulating autophagy. CK2 has been reported to be a master regulator of cellular functions in virtue of its ability to play a ‘lateral means’ of pathways intervention (12). Here, we show that effective CK2-mediated autophagy induction is achieved by the simultaneous targeting of the ERK1/2-and mTOR signaling pathways. Additional details are reported in the text.

In this study, we have shown that depletion of protein kinase CK2 leads to marked activation of autophagic cell-death and this effect is enhanced when cells are additionally treated with NCS. The effective induction of autophagy is due to CK2 action at multiple levels and results in the extensive inhibition of Raptor-mTOR complex, lack of activation of the AKT-survival pathway and marked activation of the ERK1/2 signaling cascade (Fig. 7). Further studies are necessary to shed light on the complex regulation of autophagy mediated by protein kinases, however, silencing the CK2 signal by RNA interference represents a potential therapeutic strategy for sensitizing malignant glioma cells to NCS-mediated autophagic cell death.
